# Understanding the Influence of Interface Morphology on the Performance of Perovskite Solar Cells

**DOI:** 10.3390/ma11071073

**Published:** 2018-06-25

**Authors:** Manuel Salado, Laura Calió, Lidia Contreras-Bernal, Jesus Idígoras, Juan Antonio Anta, Shahzada Ahmad, Samrana Kazim

**Affiliations:** 1 BCMaterials, Basque Center for Materials, Applications and Nanostructures, Bld. Martina Casiano, UPV/EHU Science Park, Barrio Sarriena, s/n, 48940 Leioa, Spain; manuel.salado@bcmaterials.net (M.S.); shahzada.ahmad@bcmaterials.net (S.A.); 2 Abengoa Research, Abengoa, c/Energía Solar no. 1, Campus Palmas Altas, 41014 Sevilla, Spain; calio.laura@gmail.com; 3 Area de Química Física, Universidad Pablo de Olavide, E-41013 Sevilla, Spain; lconber@upo.es (L.C.-B.); jaidileo@upo.es (J.I.); jaantmon@upo.es (J.A.A.); 4 IKERBASQUE, Basque Foundation for Science, 48013 Bilbao, Spain

**Keywords:** electron transport material, titanium oxide, charge dynamics, metal-halides perovskites

## Abstract

In recent years, organo-halide perovskite solar cells have garnered a surge of interest due to their high performance and low-cost fabrication processing. Owing to the multilayer architecture of perovskite solar cells, interface not only has a pivotal role to play in performance, but also influences long-term stability. Here we have employed diverse morphologies of electron selective layer (ESL) to elucidate charge extraction behavior in perovskite solar cells. The TiO_2_ mesoporous structure (three-dimensional) having varied thickness, and nanocolumns (1-dimensional) with tunable length were employed. We found that a TiO_2_ electron selective layer with thickness of about c.a. 100 nm, irrespective of its microstructure, was optimal for efficient charge extraction. Furthermore, by employing impedance spectroscopy at different excitation wavelengths, we studied the nature of recombination and its dependence on the charge generation profile, and results showed that, irrespective of the wavelength region, the fresh devices do not possess any preferential recombination site, and recombination process is governed by the bulk of the perovskite layer. Moreover, depending on the type of ESL, a different recombination mechanism was observed that influences the final behavior of the devices.

## 1. Introduction

During recent years, organic–inorganic halide perovskite solar cells (PSCs) have made colossal progress, competing head-to-head at lab scale with mature Silicon-based or other thin-film-based photovoltaic technologies [1]. The rapid increase in terms of power conversion efficiency (PCE) in just a few years [2,3,4] has been possible due to the exploration of rational charge selective contacts, interface optimization and the compositional engineering of perovskites [5,6]. The attractiveness of exploiting organic–inorganic halide perovskite is mainly due to its broad and high absorption co-efficient, along with its ambipolar character to transport electrons and holes [7]. Although the current record PCE values in PSCs have reached 22.7% [8], a thorough understanding of the processes involved in charge transport and recombination between layers and inside the perovskite layer is paramount.

Generally, in the case of photovoltaics, the incident photons create free carriers when they are absorbed by the semiconducting material. In the case of PSCs, these free carriers (electrons and holes) travel through the perovskite to the interface of respective charge selective contacts. Depending on the deposition techniques [9,10,11,12] and the composition of the perovskite [6,13,14] used, energetic disorder may exist mainly in the grain boundaries, which act as recombination points. In order to minimize these recombination sites, extensive effort has been made to improve the quality of the perovskite layers through compositional engineering [6], interface passivation [15,16], or processing methods such as gas-phase [17,18] or vacuum assisted deposition [19,20]. However, interfaces are vulnerable to losses due to energy-level mismatch, un-intimate contact or interfacial defects which lead to high charge accumulation, which causes a drop in the performance of the solar cell, especially in open-circuit conditions [21,22]. Thus, it is vital to understand and control charge extraction across the interfaces in order to minimize energy loss and improve device performance.

Among the diverse device structures [23] investigated to date—planar [24], mesoporous [25] and inverted [26]—mesoporous-structure-based devices employing TiO_2_ [27] and Spiro-OMeTAD [28] as the selective contacts for electrons and holes extraction respectively, yielded the best PCE and stability. Considerable research efforts have been focused on the hole selective layer (HSL), not only due to the high production cost of Spiro-OMeTAD, but also due to UV-instability and other disadvantages such as the need for dopants, which subsequently affects the performance of PSCs.

TiO_2_ is the most ubiquitous electron selective contact in mesoporous-structure-based devices. In order to improve charge extraction and reduce nanoparticle clustering, different strategies have been implemented in the past few years. For instance, Grätzel and Ahmad et al. employed Y-TiO_2_ film with the aim of improving the conductivity of TiO_2_ [29]. The results showed an increment in PCE, mostly due to better charge extraction after modifying the TiO_2_ layer. Following a similar strategy, Cojocaru et al. [30] carried out a post-treatment to the blocking TiO_2_ layer with TiCl_4_ and demonstrated an efficiency enhancement of 3% when comparing reverse- versus forward-measurement directions. In a similar fashion, Grätzel et al. [31] and Friend et al. [32] demonstrated beneficial effects when mesoporous TiO_2_ is post-treated with Li^2+^. It was argued that lithium salt can passivate the interface and can reduce the trap density. These effects led to the fabrication of devices with not only reduced hysteresis and favored electron extraction, but also improved stability.

In the recent past, electrochemical analysis such as impedance spectroscopy (IS), Intensity-modulated photocurrent spectroscopy (IMPS) and intensity-modulated photovoltage spectroscopy (IMVS) are being employed to elucidate charge recombination, accumulation and transport [33,34]. Different approaches have been proposed, from the typical perovskite device architecture where the electron selective layer (ESL) is either high temperature processed mesoporous TiO_2,_ or low temperature processed TiO_2_ in a simple planar structures that are ideal for flexible configurations. Previously, we reported [35] how the use of TiO_2_ nanocolumns affects the charge dynamics on devices, along with improvement to stability.

Herein, we report the role of TiO_2_ microstructure (3-dimensional and 1-dimensional) and thickness on charge dynamic studies in polycrystalline layers of methyl ammonium lead triiodide (MAPbI_3_). The optimization of the ESL thickness was studied by IS, to optimize charge separation and elucidate its role on charge dynamics, suggesting that, irrespective of its microstructure, an electron extraction layer with a thickness of ca. 100 nm produces better device performance in our experimental conditions.

## 2. Materials and Methods

### 2.1. Materials

All chemicals were commercial products and procured from either Sigma-Aldrich (St. Louis, MO, USA) or Acros organics (Geel, Belgium) and were employed as such. 2,2′,7,7′-tetrakis(*N*,*N*-di-p-methoxyphenyamine)-9,9-spirobifluorene (Spiro-OMeTAD) was acquired from Merck KGaA (Darmstadt, Germany). Methylamine iodide, CH_3_NH_3_I (MAI) was bought from Greatcells Solar (Queanbeyan, New South Wales, Australia) and PbI_2_ were bought from TCI (Tokyo, Japan). Chlorobenzene and anhydrous DMSO were obtained from Sigma-Aldrich (St. Louis, MO, USA). Acetone, acetonitrile solvents were bought from Acros organics.

### 2.2. Electron Selective Layer (ESL) Deposition

Vertically aligned nanocolumns with a thickness of 100 or 200 nm were prepared at room temperature by physical vapor deposition at oblique incidence (GLAD-PVD) in an electron beam evaporator (Advanced Products and Technologies GmbH, Nurtingen, Germany), as described in previous publications [35,36]. The deposition time was controlled to achieve two different nanostructured thicknesses, i.e., 100 or 200 nm. In case of TiO_2_ mesoporous layer (30NRD, Greatcells Solar, Queanbeyan, Australia), to obtain different thicknesses, mesoporous stock solutions with different dilution (1:7 and 1:3.5 *w*/*w*) were prepared and spun-coated at 4000 rpm for 20 s. For the fabrication of planar devices, no such layer was used. All the ESL-based substrates were then annealed up to 450 °C in a progressive manner for 2 h.

### 2.3. Device Preparation

To fabricate the PSCs, laser-etched FTO-coated glass (TEC15, Pilkington, Tokyo, Japan) was used. Prior to use, the electrodes were cleaned using Hellmanex solution and washed with deionized water. Subsequently, they were ultrasonicated for 25 min at 60 °C in acetone, 2-propanol, and finally dried using nitrogen blow. The compact layer (hole-blocking layer) was made up of TiO_2_, deposited by spray pyrolysis at 450 °C by diluting 1 mL of titanium diisopropoxide bis(acetyl acetonate) precursor solution (75% in 2-propanol, Sigma-Aldrich) in 19 mL of pure ethanol and dry air as carrier gas. These substrates were then kept for a further 30 min at 450 °C. Once the TiO_2_ deposited electrodes attained room temperature, they were immersed in a 0.02 M TiCl_4_ solution in deionized water at 70 °C for 30 min to form a homogeneous layer. After this treatment, the electrodes were washed by using deionized water and annealed at 500 °C for 30 min, and then allowed to cool down to room temperature.

For deposition of the perovskite layer, 1.2 M of PbI_2_ and MAI precursor solutions (1:1 ratio), in DMSO were prepared inside an argon-filled glovebox (H_2_O level: <1 ppm and O_2_ level: <10 ppm) and left for stirring overnight at 80 °C. The perovskite layer was deposited using the so called one-step method followed by solvent engineering; for this the precursor solution was spun-coated on top of the mesoporous layer at 1000 rpm for 10 s and then 6000 rpm for 20 s. Chlorobenzene was dripped during the second stage of spinning process. Following this the electrodes were transferred to a hotplate and annealed at 100 °C for 1 h to allow the formation of perovskites. The hole transport layer was then deposited on top of the perovskites; for this 35 μL of a Spiro-OMeTAD solution was then spun-coated at 4000 rpm for 20 s. Spiro-OMeTAD material (70 mM) was dissolved in 1 mL of chlorobenzene and standard additives such as 17.5 μL of a lithium bis(trifluoromethylsulphonyl)imide (LiTFSI) stock solution (520 mg of LiTFSI in 1 mL of acetonitrile), 21.9 μL of a FK209 (Tris(2-(1H-pyrazol-1-yl)-4-tert-butylpyridine) cobalt(III)Tris(bis(trifluoromethylsulfonyl)imide))) stock solution (400 mg in 1 mL of acetonitrile), and 28.8 μL of 4-tert-butylpyridine (*t*-BP). The device was finished by depositing 80 nm of gold as a cathode layer, using thermal evaporation under a vacuum level between 10^−6^ to 10^−5^ torr.

For IS measurements, light sources were provided using red (λ = 635 nm) and blue (λ = 465 nm) light emitting diodes (LEDs) in a broad range of DC light intensities. By doing so, the devices could be probed on two different optical penetrations [37]. To record the impedance spectra, a response analyzer module ((PGSTAT302N/FRA2, Metrohm Autolab, Utrecht, Netherlands) was employed, and 20 mV perturbation in the 10^7^–10^−2^ Hz range was applied for the measurements. The voltage drop (arising due to series resistance) was avoided by performing the measurements at the open-circuit potential, and the Fermi level (linked to the open-circuit voltage) was fixed by the DC (bias) illumination intensity. The different response under blue and red light was compensated by monitoring all parameters and was plotted as a function of the open-circuit potential generated by individually bias light.

## 3. Results and Discussion

**Figure 1:**
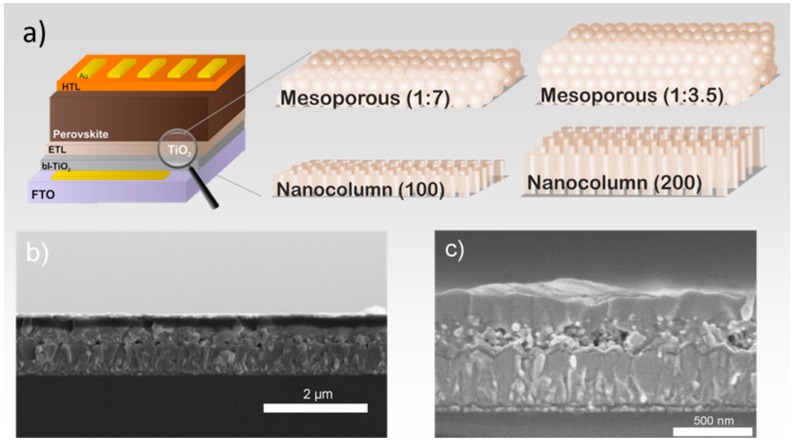
(**a**) Cross-sectional device scheme and electron selective layer (ESL) used, (**b**) scanning electron microscopy (SEM) image of PSC with 1:7 TiO_2_ mesoporous structure and (**c**) SEM image of PSC with 1:3.5 TiO_2_ mesoporous structure.

**Figure 2:**
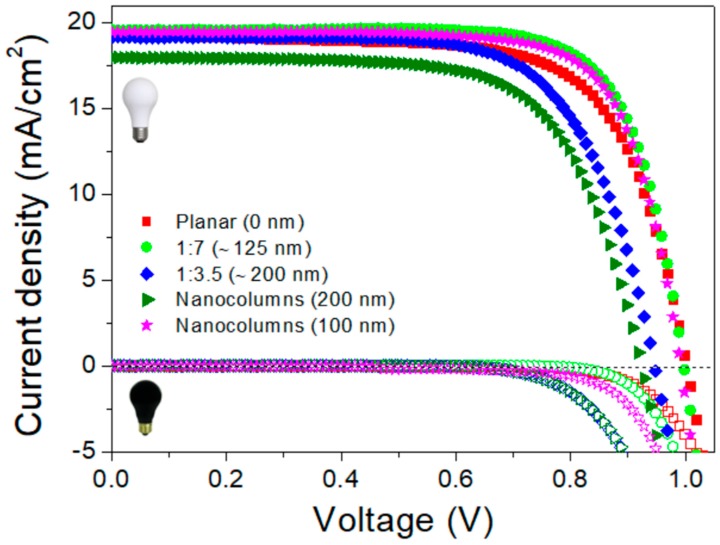
Current density-voltage (*J-V*) characteristics of the best performing PSCs measured in dark and under illumination at AM 1.5G, 100 mW/cm^2^ in reverse bias scans performed at 100 mV s^−1^, for different electron selecting layers. Hollow symbols indicate the dark current of the different ESL-based devices.

Figure 1a represents a schematic of the fabricated solar cell, in which the different layers (FTO/bl-TiO_2_/mesoporous or nanocolumns TiO_2_/CH_3_NH_3_PbI_3_/Spiro-OMeTAD/Au) can be visualized. In order to observe morphological difference of the interface between the absorber layer and the electron extraction layer, scanning electron microscopy (SEM, Hitachi, Tokyo, Japan) experiments were performed. Figure 1b,c represents the cross-sectional SEM images of the fabricated devices having different thicknesses of mesoporous structure. The thickness of the mesoporous structure was tuned by varying the dilution rate of the TiO_2_ paste; for example, the more diluted TiO_2_ paste led to a thin layer (Figure 1 and Table S1). In case of TiO_2_ nanocolumns, the length of columns was varied by increasing the deposition time. Considering the values in Table S1, the total thickness of photoactive layer (mesoporous & perovskite capping layer) was c.a. 300 nm. It can be seen from the figures that the thickness of the perovskite capping layer was also altered when the dilution rate was modified, having a strong influence in the series resistance of the device (Figure S1). The aim of using different microstructures (3D and 1D) was to optimize the effective thickness for ideal perovskite infiltration and electron extraction. According to the current density-voltage (*J-V*) curves (Figure 2), the best configuration was found to be 1:7, which allowed a thickness of around 100 nm (Table S1).

TiO_2_ nanostructure and its thickness can influence the properties of the layer deposited atop of it. Earlier, we have presented the cross-sectional image of 1-D TiO_2_ nanocolumns (100 and 200 nm) [35]. To study the role of TiO_2_ microstructure effects on the band gap of the perovskite, we calculated the optical band gap of devices having different ESL configurations using the Tauc plot method. The absorption coefficient (*α*) was calculated from measured absorbance spectra (Figure S2a) and a Tauc plot (*αhν*)^2^ vs. *hν* was used to calculate the optical direct band gap as shown in Figure S2b. The linear extrapolation of this curve to intercept the horizontal hν axis gives the value of band gap (~1.59 eV).

Figure 2 illustrates the *J-V* curves of fabricated PSCs using different ESL under standard conditions (100 mW·cm^−2^—AM 1.5 illumination) at room temperature. Table 1 summarizes the different photovoltaic parameters of the best-performing devices for each configuration, while their statistical data are listed in Table S2. As expected, the thickness of the ESL was found to influence the photovoltaic performance.

A lower value of *V*_oc_ was noted (*V*_oc_ = 930 mV) with a thicker TiO_2_ layer (nanocolumns-200 nm and nanoparticles-1:3.5), in comparison to the thinner TiO_2_ ESLs (nanocolumns-100 nm and nanoparticles-1:7), or planar devices (0 nm). Although no significant changes were observed in terms of the *J*_sc_, and regardless of the nanostructure and thickness of the ESLs, all devices gave *J*_sc_ in the range of 18–19 mA·cm^−2^. These results are in line with the absorbance spectra obtained for the different configuration (Figure S2). Devices fabricated with mesoporous layer (1:7, Ø30 nm) and 100 nm nanocolumns yielded best PCE, as well as lower series resistance (Figure S1).

In PSCs, the series resistance (*R*_s_) is known as a crucial factor which can affects the device fill factor, and the *R*_s_ depends not only on bulk resistance of the photoactive layer, but also on metallic contacts and contact resistance at each interface. The *R*_s_ of the devices (obtained from the software by fitting the *J-V* curve) based on 100 nm TiO_2_ nanocolumns and thin mesoporous layers (1:7 dilutions, thickness around 125 nm) show a lower *R*_s_ value than the 200 nm nanocolumns and thick mesoporous layer (1:3.5). The reduced value of *R*_s_ agrees with the enhanced FF for thin TiO_2_ ESLs (mp-1:7 and 100 nm NC), irrespective of their morphology.

Dissimilarities in device behavior can be also analyzed from the dark-current measurements (represented by the hollow symbols in Figure 2). It is known that the *V*_oc_ is strongly related to the onset voltage point of the device measured in the dark under a forward bias, and delay in the onset voltage point of the device will result in high *V*_oc_. Devices prepared with mesoporous TiO_2_ layer (1:7, Ø30 nm), 100 nm TiO_2_ nanocolumns and planar devices exhibit comparatively lower dark current and late switch-on of the devices under forward bias than the thicker mesoporous (1:3.5, Ø30 nm), and 200 nm nanocolumns. The delay in the onset of the dark current for the abovementioned devices is well supported, with comparatively high values of *V*_oc_ (Table 1), indicating reduced charge recombination rates in thinner ESLs, which will be further demonstrated by impedance measurements in the next section.

However, an ESL based on non-planar structure helps to minimize the hysteresis. As shown in Table S3, all non-planar devices show a hysteresis index (HI) of around 0.1–0.2, whereas a value of 0.48 was found for planar devices.

HI values for the MAPbI_3_ based PSCs using different ESLs were calculated (Table S3) along with the *J-V* parameters in forward and reverse directions for the best-performing device. Hysteresis behavior has been attributed to several causes, such as ionic migration, charge accumulation at selective contacts interfaces or distortion of the octahedral structure, or a combination of all of these factors. Recently, research direction has been focused on the slow processes occurring in the PSCs to study possible cause of hysteresis. The charge accumulation at the TiO_2_/perovskite interface coupled with ionic migration may cause this phenomenon [23,30,38].

In order to unravel the charge dynamics depending on the different ESLs, IS experiments were performed. This technique provides information about various internal processes occurring in working devices in different operation conditions and offers the possibility of analyzing interfacial and bulk processes separately. The resistive and capacitive processes occurring in the device are related to bulk and interfacial processes such as charge transport, accumulation, recombination and ion-mediated processes. Charge accumulation and ion migration can be probed by IS, and both processes are possibly interconnected and contribute to the hysteresis behavior. Charge imbalance also promotes an intrinsic instability, along with an irrational interface, both of which can accelerate the degradation of the fabricated devices. In our case, we have observed that devices with higher thickness (non-planar devices) of the ESL exhibited smaller values of HI than planar devices.

**Figure 3:**
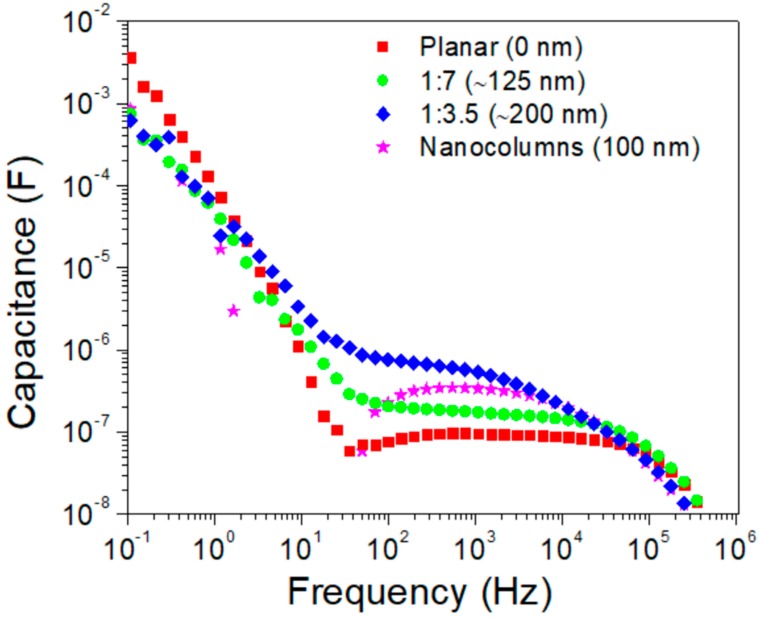
Frequency dependent apparent capacitance for PSCs using different electron selective layer at 0.9 V.

Figure 3 shows the apparent capacitance versus frequency plots, illustrating the different processes depending on the range of frequency. Low-frequency processes could be related to hysteresis behavior and thus we studied the variation of the low-frequency capacitance as a function of photo-voltage (Figure S3). The value of slope (V^−1^) close to 22 indicates the existence of an accumulation regime. Our results suggest that planar configuration presents a higher slope than non-planar-based structure, suggesting a possible higher charge accumulation at the interface.

**Figure 4:**
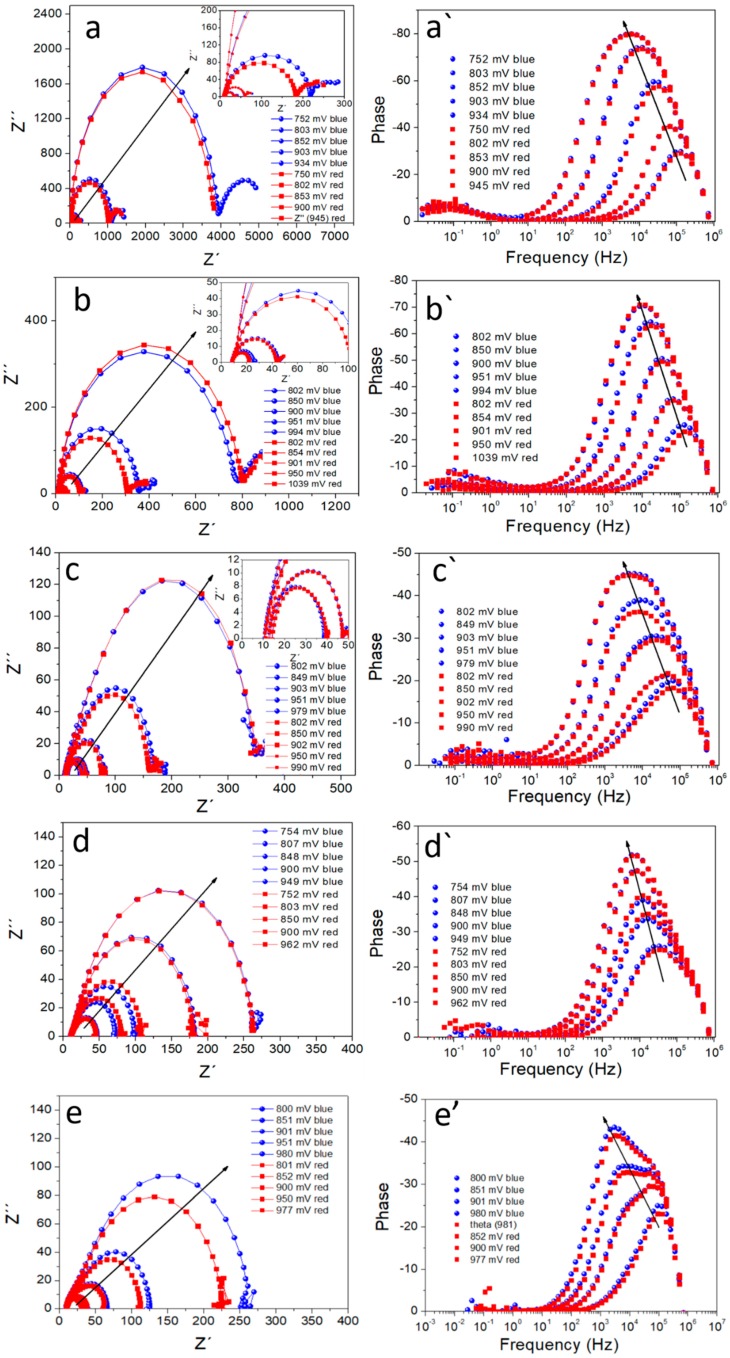
Nyquist (**a**–**e**) and Bode plots (**a`**–**e`**) at different applied voltages for CH_3_NH_3_PbI_3_ perovskite solar cells using two excitation wavelengths, λ_blue_ = 465 nm and λ_red_ = 635 nm. Different configurations are labeled as follows: (**a**,**a`**) planar, (**b**,**b`**) mesoporous 1:7, (**c**,**c`**) mesoporous 1:3.5, (**d**,**d`**) nanocolumns 100 nm and (**e**,**e`**) nanocolumns 200 nm. Arrows indicate the evolution of the measurements when we measured from *V*_OC_ to lower values.

**Figure 5:**
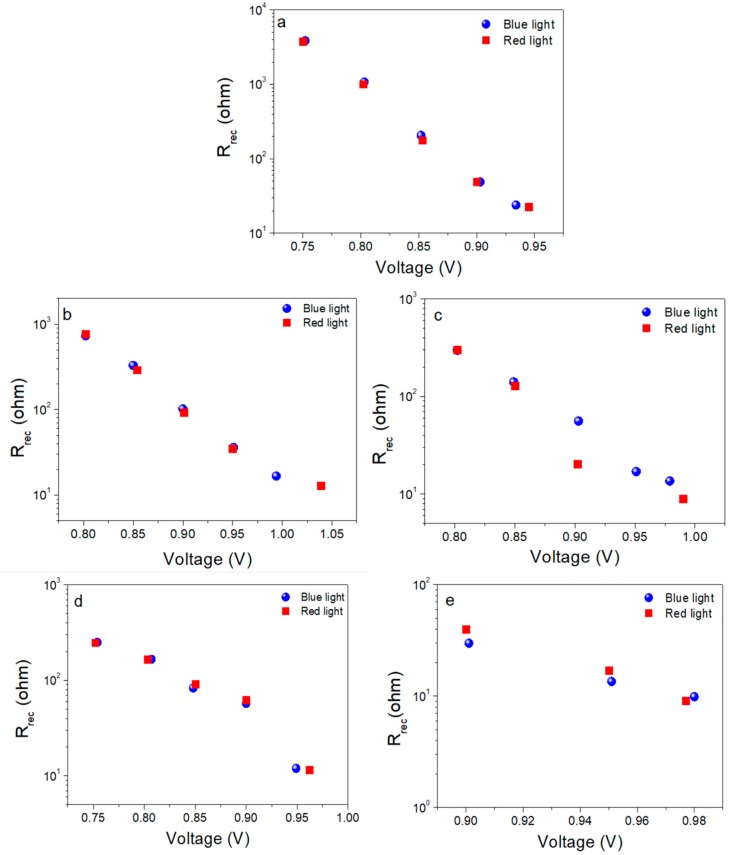
Recombination resistance plots (**a**–**e**) at different applied voltages for CH_3_NH_3_PbI_3_ perovskite using the two excitation wavelengths, λ_blue_ = 465 nm and λ_red_ = 635 nm. Different configurations are labeled as follows: (**a**) planar, (**b**) mesoporous 1:7, (**c**) mesoporous 1:3.5, (**d**) nanocolumns 100 nm and (**e**) nanocolumns 200 nm.

Furthermore, we undertook experiments to measure the charge dynamics that determine the functioning of PSCs. For this, we performed IS measurements with two different excitation wavelengths (blue and red) [37]. Based on the different penetration depths, which provoke a dissimilar charge generation profile with the use of two illumination wavelengths, the main purpose of this characterization was to detect in-homogeneities or the existence of a favored recombination region inside the solar device. By implementing this, we can study recombination processes occurring close to the electron (blue) and hole (red) in a more homogeneous way, as we will create different charge-generation profiles. As shown in Figure 4, two arcs with different characteristic frequencies can be distinguished for all the analyzed samples; one on the low frequency (LF) region (0.1–10 Hz), and one in the high-frequency (HF) region (10^2^–10^5^ Hz). The semicircle at high frequencies can be directly linked to the recombination kinetics in the devices, while the semicircle at low frequencies, although also indirectly affected by recombination, is related to slow processes such as ionic migration or charge accumulation at the interfaces. We have found that IS measurements under red and blue light coincide with each other (Figure 4). By fitting the impedance response under blue and red illumination for MAPbI_3_/Spiro devices to a simple -R_S_-(R_LF_,-CPE_1_)-(R_HF_,-CPE_2_)-equivalent circuit, the HF (recombination) resistance can be extracted (Figure 5). In this analysis, a constant phase element (CPE) replaces an ideal capacitor in order to obtain a better fitting. The resulting R_HF_ values under red and blue light excitation also coincide quite well, confirming that fresh samples do not possess any preferential recombination site. This fact points toward recombination processes occurring in the bulk of the perovskite layer, as previously reported [37].

**Figure 6:**
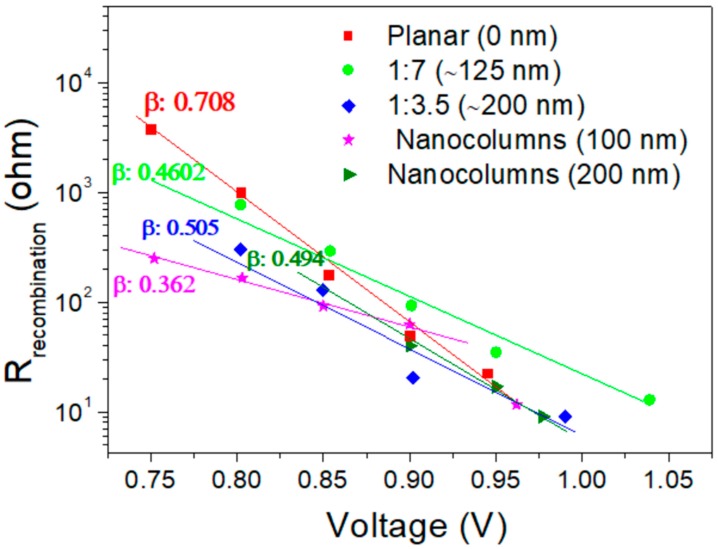
The representative recombination resistances (R_recombination_) of the different TiO_2_ structures samples determined from IS under different applied bias voltages irradiated with red light.

Figure 6 shows the R_HF_ values under red light excitation for all samples studied. Different slopes were identified depending on the ESL configuration. Previous studies reported the relationship between these slopes and the predominant recombination mechanism inside the solar device (β parameter in Figure 6) [37,39]. Our results (Table S4), suggest that the planar configuration presents a different or mixed recombination mechanism (*n* = 1/β = 1.41) with respect to the trap-limited recombination mechanism observed in the others studied configuration (*n* = 1/β ~ 2). Considering the consistency between the recombination resistance data obtained with red and blue optical penetration profiles, which points toward bulk recombination, the different ideality factors obtained suggest that electrical features of perovskite depend on the nature of the ESL on which it is deposited. Even though the band gap of the perovskite remains unaltered, the crystallization process might be different, which will affect the transport and recombination features inside the active perovskite layer. It is well reported that the capacitance in the high-frequency range of the PSC is mostly governed by the geometrical component, which can be expressed as following; *C*_g_ = ε∙ε_0_∙A/d. Here ε is the dielectric constant of the perovskite, ε_0_ is the permittivity in vacuum, A is the effective contact area and d is the thickness of the absorber layer. The value of capacitance extracted from the high-frequency component (Figure S4) was constant on bias voltage; this is in accordance with its geometrical nature.

The role of ESL in devices’ degradation was monitored, by recording *J-V* measurements and incident photon-to-electron conversion efficiencies (IPCE) after 30 days (Figure S5a,b). We found that non-planar devices were more stable than planar ones. IPCE measurements of aged devices showed a systematic depletion of the photo generation in the full-wavelength range. Furthermore, following the methodology of previous works [37,39], we also measured the open-circuit voltage (*V*_oc_) as a function of temperature (T in K) in order to extract the band gap of aged devices. Figure S6 represents the open-circuit voltage versus temperature curve of aged devices (stored under humid conditions for 30 days), and fits well into the straight line. The activation energy—which gives information about dominant recombination pathway—was estimated by extrapolation of the data to T = 0 K. It can be observed that the calculated band gaps show increment with respect to fresh devices (*E*_g_ calculated by means of the absorbance measurements). These results are in accordance with the previous published results [37].

We propose that the increase in the band gap can be attributed to the emergence of lead iodide crystal due to a partial degradation of the perovskite material which can be located at the TiO_2_/perovskite interface. It is worth noting that nanocolumns (100 nm) and mesoporous (1:7) present the lower deviation in *E*_g_ with respect to the initial values, indicating their better stability under aging. In the case of planar devices, a decrease in the band gap was noted. The decrease in *V*_oc_, can be attributed to the formation of defect sites, which further induce new recombination sites.

## 4. Conclusions

Interfaces at the perovskite perform a crucial role in charge separation and transport for solar cells fabrication. Electron selective contacts, having different microstructure (3-dimensional and 1-dimensional) and thickness of porous TiO_2_ layer, were fabricated. The thickness and morphology of the electron selecting layer influenced the device performance and stability, possibly by affecting the perovskite crystallization process or the charge transfer at the interface. In the case of planar devices, a higher hysteresis index was observed, while both mesoporous (3D) and nanocolumns layer (1D) with 100 nm thickness gave good performance and stability.

By using impedance spectroscopy measurements, the samples were illuminated with different wavelengths (blue and red), and similar recombination resistance data were obtained irrespective of wavelength region, suggesting that the recombination process at open circuit occurs in the bulk of the perovskite layer. However, depending on the type of electron selective layer deposited, a different recombination path was observed as demonstrated by a different value of ideality factor, suggesting that electrical features of a perovskite layer depend on the nature of the ESL on which they are deposited. This work contributes to an understanding of the role of electron selective contact and its bearing on charge kinetics in perovskite solar cells.

## Figures and Tables

**Table 1:** *J-V* characteristics parameters of best-performing device based on MAPbI_3_ perovskite with different electron selecting layer. * The value in parenthesis depicts the thickness used.

Device Configuration *	*V*_oc_ (mV)	*J*_sc_ (mA/cm^2^)	Fill Factor (%)	Efficiency (%)
Planar (0 nm)	1000	19.28	69.04	13.45
mp-1:3.5 (~200 nm)	940	19.15	68.60	12.42
mp-1:7 (~125 nm)	990	19.55	75.07	14.61
Nanocolumns (200 nm)	920	17.94	68.07	11.26
Nanocolumns (100 nm)	990	19.44	74.47	14.35

## Supplementary Materials

The following are available online at http://www.mdpi.com/1996-1944/11/7/1073/s1, Figure S1: Series resistance under 1 sun-illumination for different electron transport structures with reverse scans performed at 0.1 V s^−1^, Figure S2: (a) Absorption spectra of CH_3_NH_3_PbI_3_ perovskite infiltrated in different TiO_2_ structures. (b) Tauc plot to estimate optical band gap for perovskite films in the presence of TiO_2_ mesoporous or nanocolumnar structures having different thickness, Figure S3: Low frequency capacitance as a function of open circuit voltage of the PSCs using TiO_2_ electron transport layer, Figure S4: Geometric capacitance at different applied voltages for CH_3_NH_3_PbI_3_ perovskite using two excitation wavelengths λ_blue_ = 465 nm and λ_red_ = 635 nm, Figure S5: J-V and IPCE measured after 30 days at humid conditions; (a) dark and under white light illumination, (b) IPCE and calculated integrated short circuit current, Figure S6: Open-circuit voltage as a function of temperature for PSCs with different TiO_2_ configurations under white light intensity of 14.15 W/m^2^, Table S1: Layer thicknesses extracted from SEM images of MAPbI_3_ perovskite solar cells using different thickness of TiO_2_ ESL, Table S2: Statistical data of photovoltaics parameters of MAPbI_3_-based PSCs, Table S3: J-V characteristic parameters values from the reverse and forward scan directions and calculated hysteresis index of the different ESL configurations, Table S4: Ideality factors of the perovskite solar cells acquired from impedance measurement using different electron transport configuration.
