# Supplementary material for: Understanding the Influence of Interface Morphology on the Performance of Perovskite Solar Cells
Source: Materials (Basel). 2018 Jun 25;11(7):1073. doi: 10.3390/ma11071073 (PMC6073852; doi:10.3390/ma11071073)
Supplement: Supplementary file 1 [file materials-11-01073-s001.pdf]

materials

Understanding the Influence of Interface Morphology on the Performance of Perovskite Solar Cells

Manuel Salado [1,†], Laura Calió [2,†], Lidia Contreras-Bernal [3], Jesus Idígoras [3], Juan Antonio Anta [3], Shahzada Ahmad [1,2,4] and Samrana Kazim [1,2,*]

1 BCMaterials, Basque Center for Materials, Applications and Nanostructures, Bld. Martina Casiano, UPV/EHU Science Park, Barrio Sarriena , s/n, 48940, Leioa, Spain; manuel.salado@bcmaterials.net (M.S.); shahzada.ahmad@bcmaterials.net (S.A.)
2 Abengoa Research, Abengoa, c/Energía Solar no. 1, Campus Palmas Altas, 41014, Sevilla, Spain; calio.laura@gmail.com (L.C.);
3 Area de Química Física, Universidad Pablo de Olavide, E-41013, Sevilla, Spain; lconber@upo.es (L.C.-B.); jaidileo@upo.es (J.I.); jaantmon@upo.es (J.A.A.)
4 IKERBASQUE, Basque Foundation for Science, 48013, Bilbao, Spain
* Correspondence: samrana.kazim@bcmaterials.net
† These authors contributed equally to this work.

Supporting information

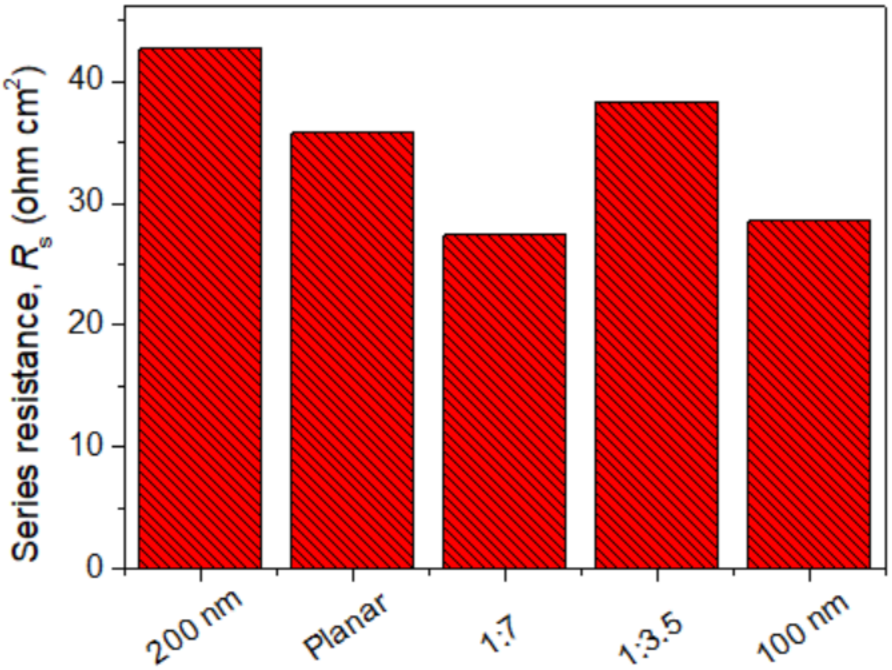

Figure S1. Series resistance under 1 sun-illumination for different electron transport structures with reverse scans performed at 0.1 V s^{-1}.

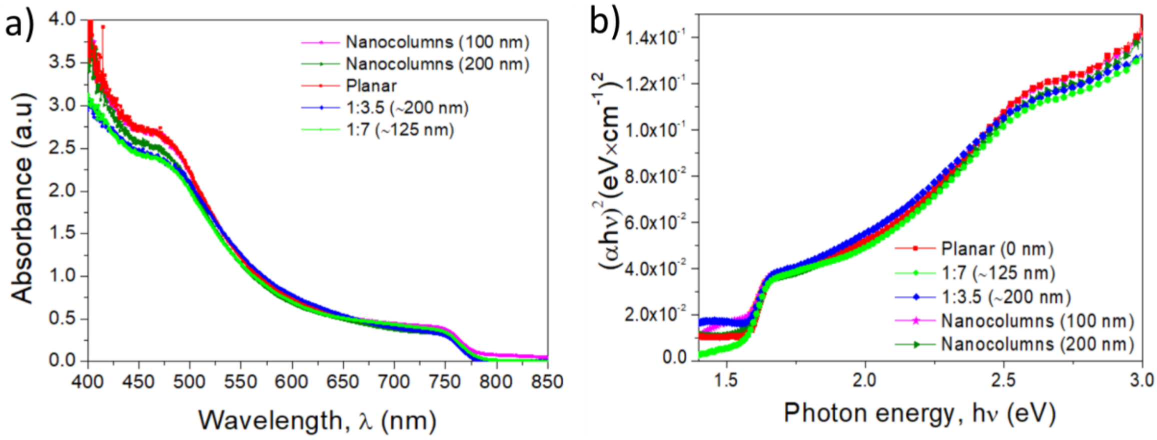

Figure S2. a) Absorption spectra of $CH_3NH_3PbI_3$ perovskite infiltrated in different TiO_2 structures and b) Tauc plot to estimate optical band gap for perovskite films in the presence of TiO_2 mesoporous or nanocolumnar structures having different thickness. The band gap remains largely unchanged and shows value of 1.59 eV.

Table S1. Layer thicknesses extracted from SEM images of $MAPbI_3$ perovskite solar cells using different thickness of TiO_2 ESL

Layer/Dilution 30NRD	Blocking Layer (nm)	Mesoporous (nm)	Perovskite (nm)
Planar	Around 40-50	0	300
1:7		125.1±16.3	147.7±10.6
1:3.5		197.57±20	123.3±9.64

Table S2. Statistical data of PV parameters of $MAPbI_3$ based PSCs**

Device configuration	*V*oc (V)	*J*sc (mA/cm²)	Fill Factor (%)	Efficiency (%)
Planar(0 nm)	0.98 ± 0.008	19.22 ± 0.15	64.09 ± 7.14	12.26 ± 1.3
1:3.5 (~200 nm)	0.92 ± 0.01	17.72 ± 0.96	67.67 ± 1.22	11.09 ± 0.92
1:7 (~125 nm)	0.98 ± 0.01	19.57 ± 0.53	69.94 ± 5.34	13.5 ± 0.88
Nanocolumns (200 nm)	0.90 ± 0.02	18.0 ± 0.06	67.24 ± 1.65	10.94 ± 0.5
Nanocolumns (100 nm)	0.95 ± 0.02	18.7 ± 0.66	70.36 ± 3.98	12.62 ± 1.43

**Each value is derived from 4 cells made in a batch of 20, Samples were measured with a mask of 0.16 cm²

Table S3. *J*-*V* characteristic parameters values from the reverse and forward scan directions and calculated Hysteresis index of the different ESL configurations.

Configuration	*V*oc	*J*sc	FF	PCE	Hysteresis index (HI)
Planar (0 nm)	0.99	19.39	58.7	11.36	0.48
	0.97	19.23	35.26	6.63	
Mesoporous 1:7 (125 nm)	0.99	19.63	75.32	14.67	0.11
	0.928	19.55	59.69	11.44	
Mesoporous 1:3.5 (200 nm)	0.948	19.22	68.7	12.52	0.21
	0.936	19.17	63.01	11.32	
Nanocolumns (100 nm)	0.976	19.42	72.86	13.82	0.19
	0.95	19.24	59.35	10.87	
Nanocolumns (200 nm)	0.918	17.98	68.65	11.4	0.20
	0.899	17.91	57.46	9.25	

Table S4. Ideality factors of the perovskite solar cells obtained from impedance measurement using different electron transport configuration.

Electron transport configuration	β-parameter	Ideality factor (n = 1/ β)
Planar (0 nm)	0.708	1.41
Mesoporous 1:7 (125 nm)	0.4602	2.17
Mesoporous 1:3.5 (200 nm)	0.505	1.98
Nanocolumns (100 nm)	0.362	2.76
Nanocolumns (200 nm)	0.494	2.02

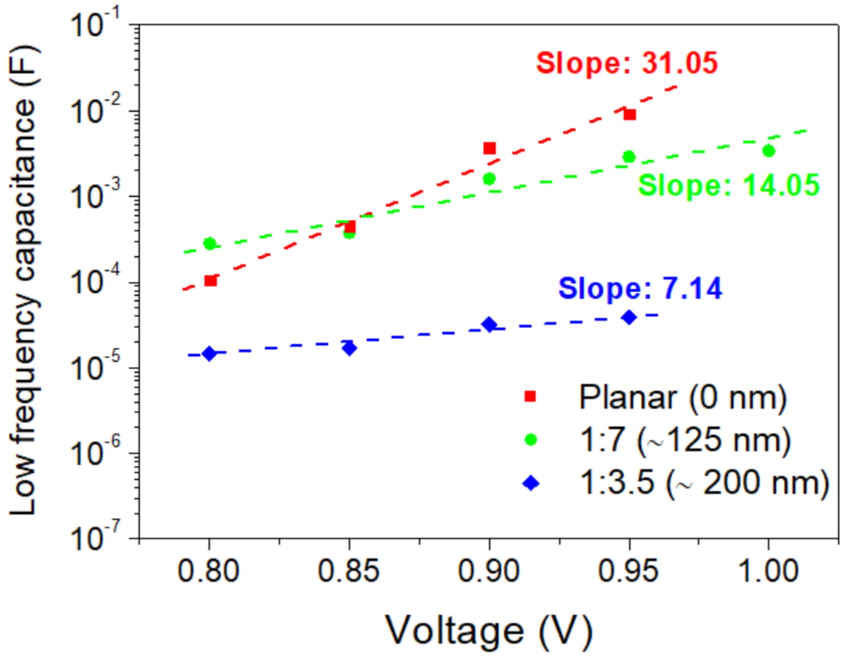

Figure S3. Low frequency capacitance as a function of open circuit voltage of the PSCs using TiO_2 electron transport layer with different thickness. Slope in V^{-1} is extracted from the linear regime.

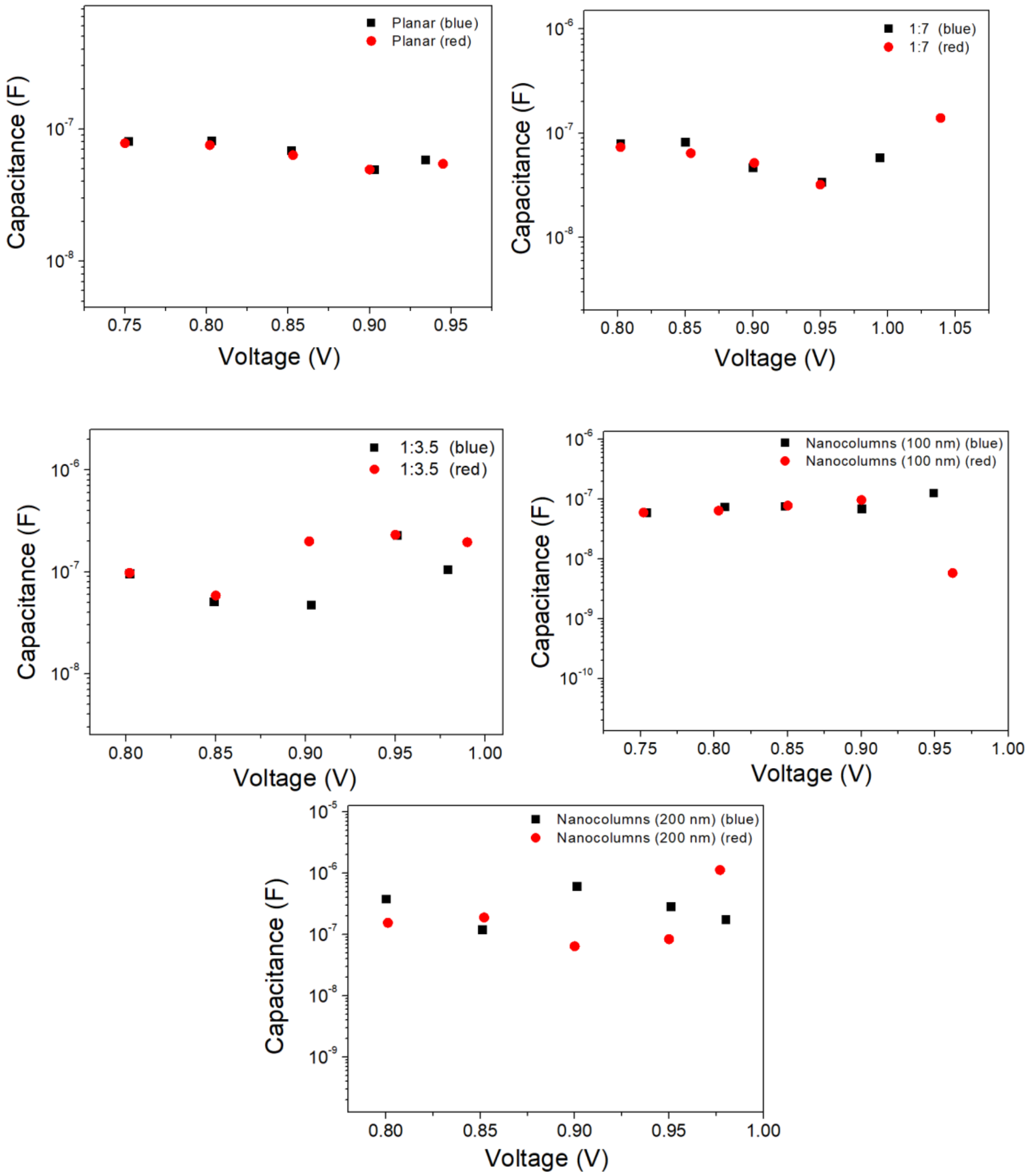

Figure S4. Geometrical capacitance at different applied voltages for $CH_3NH_3PbI_3$ perovskite using two excitation wavelength λ_{blue} = 465 nm and λ_{red}= 635 nm.

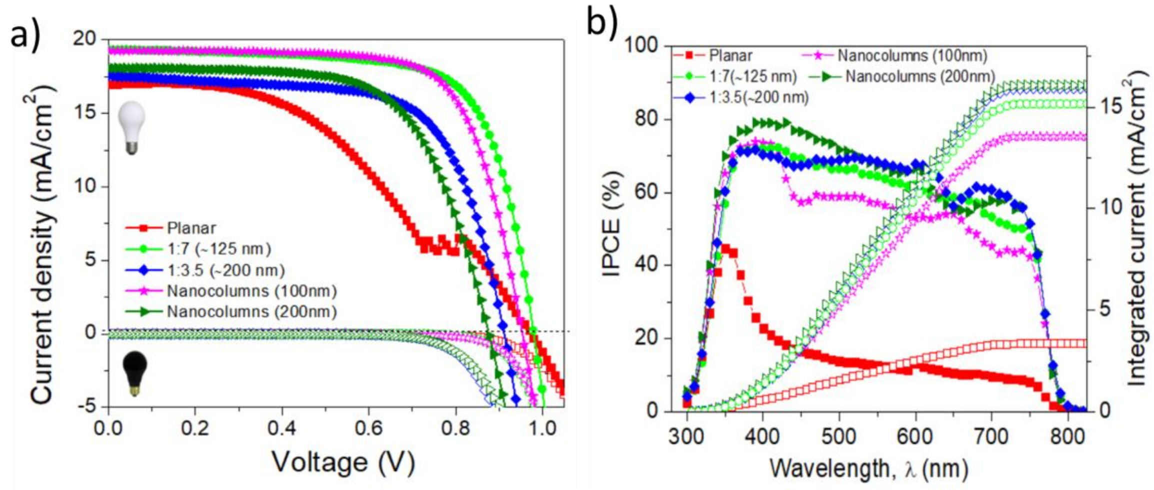

Figure S5. *J-V* and IPCE measured after 30 days in humid conditions; a) *J-V* curves measured in dark (hollow symbols indicate the dark current of the different ESL based devices) and under white light 1 sun illumination (reverse scans performed at 0.1 mV s^{-1}), b) IPCE measured for different electron transport structures after 30 days in humid conditions (hollow symbols indicate the calculated integrated short circuit current of corresponding IPCE curve.

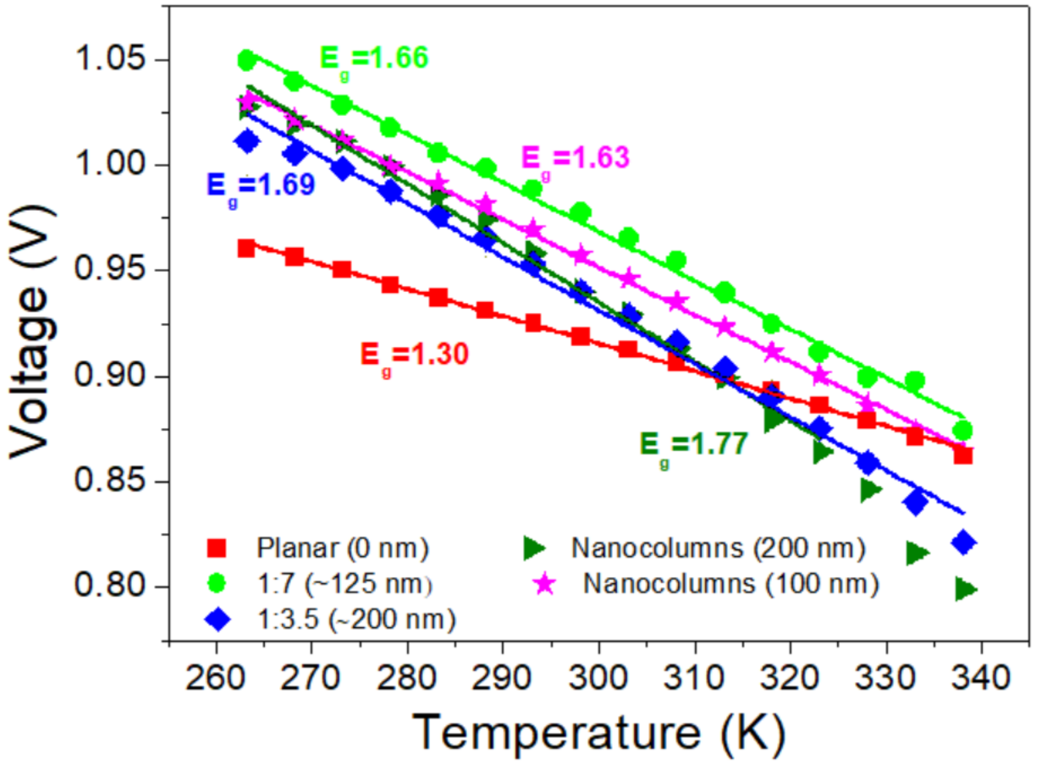

Figure S6. Open-circuit potential as a function of temperature for PSCs fabricated with different TiO_2 configurations described in Table 1 under white light intensity of 14.15 W/m^2.
